# A nematode-derived, mitochondrial stress signaling-regulated peptide exhibits broad antibacterial activity

**DOI:** 10.1242/bio.058613

**Published:** 2021-05-20

**Authors:** Madhab Sapkota, Mohammed Adnan Qureshi, Siraje Arif Mahmud, Yves Balikosa, Charlton Nguyen, Joseph M. Boll, Mark W. Pellegrino

**Affiliations:** Department of Biology, University of Texas Arlington, Arlington, 76019 Texas, USA

**Keywords:** CNC-4, Caenacins, Antimicrobial peptide, Mitochondria, Mitochondrial UPR, Stress response, Innate immunity

## Abstract

A dramatic rise of infections with antibiotic-resistant bacterial pathogens continues to challenge the healthcare field due to the lack of effective treatment regimes. As such, there is an urgent need to develop new antimicrobial agents that can combat these multidrug-resistant superbugs. Mitochondria are central regulators of metabolism and other cellular functions, including the regulation of innate immunity pathways involved in the defense against infection. The mitochondrial unfolded protein response (UPR^mt^) is a stress-activated pathway that mitigates mitochondrial dysfunction through the regulation of genes that promote recovery of the organelle. In the model organism *Caenorhabditis elegans*, the UPR^mt^ also mediates an antibacterial defense program that combats pathogen infection, which promotes host survival. We sought to identify and characterize antimicrobial effectors that are regulated during the UPR^mt^. From our search, we discovered that the antimicrobial peptide CNC-4 is upregulated during this stress response. CNC-4 belongs to the caenacin family of antimicrobial peptides, which are predominantly found in nematodes and are known to have anti-fungal properties. Here, we find that CNC-4 also possesses potent antimicrobial activity against a spectrum of bacterial species and report on its characterization.

## Abstract (precis)

**Summary:** The caenacin antimicrobial peptide CNC-4 is regulated by a mitochondrial recovery pathway and exhibits broad antibacterial activity.

## INTRODUCTION

Treatment of multidrug-resistant pathogenic infections has become a significant challenge in recent years, demonstrated by prolonged hospital stays, higher medical costs and increased mortality (Dadgostar, 2019). There is a considerable need, therefore, to discover and develop new antimicrobial therapeutics to combat these potentially difficult-to-treat infections. In addition, identification and characterization of host antimicrobial pathways may unveil new strategies in the fight against challenging pathogenic infections.

Mitochondria are essential organelles involved in multiple cellular functions including energy production via oxidative phosphorylation (OXPHOS), amino acid metabolism, and programmed cell death. In addition, mitochondria are important mediators of innate immunity, the host's first line of defense against pathogen infection. For example, mitochondrial reactive oxygen species (ROS) that are produced as a by-product of OXPHOS can activate innate immune pathways such as NF-κB (Chandel et al., 2000), as well as acting directly as an anti-microbial agent (West et al., 2011). Mitochondria are also involved in the activation of the inflammasome via cardiolipin, a phospholipid present in mitochondrial membranes (Iyer et al., 2013), as well as mitochondrial ROS (Zhou et al., 2011). And, mitochondrial DNA itself can act as a damage-associated molecular pattern in the activation of innate immunity following Toll-like-receptor recognition (Collins et al., 2004).

**Fig. 1.**
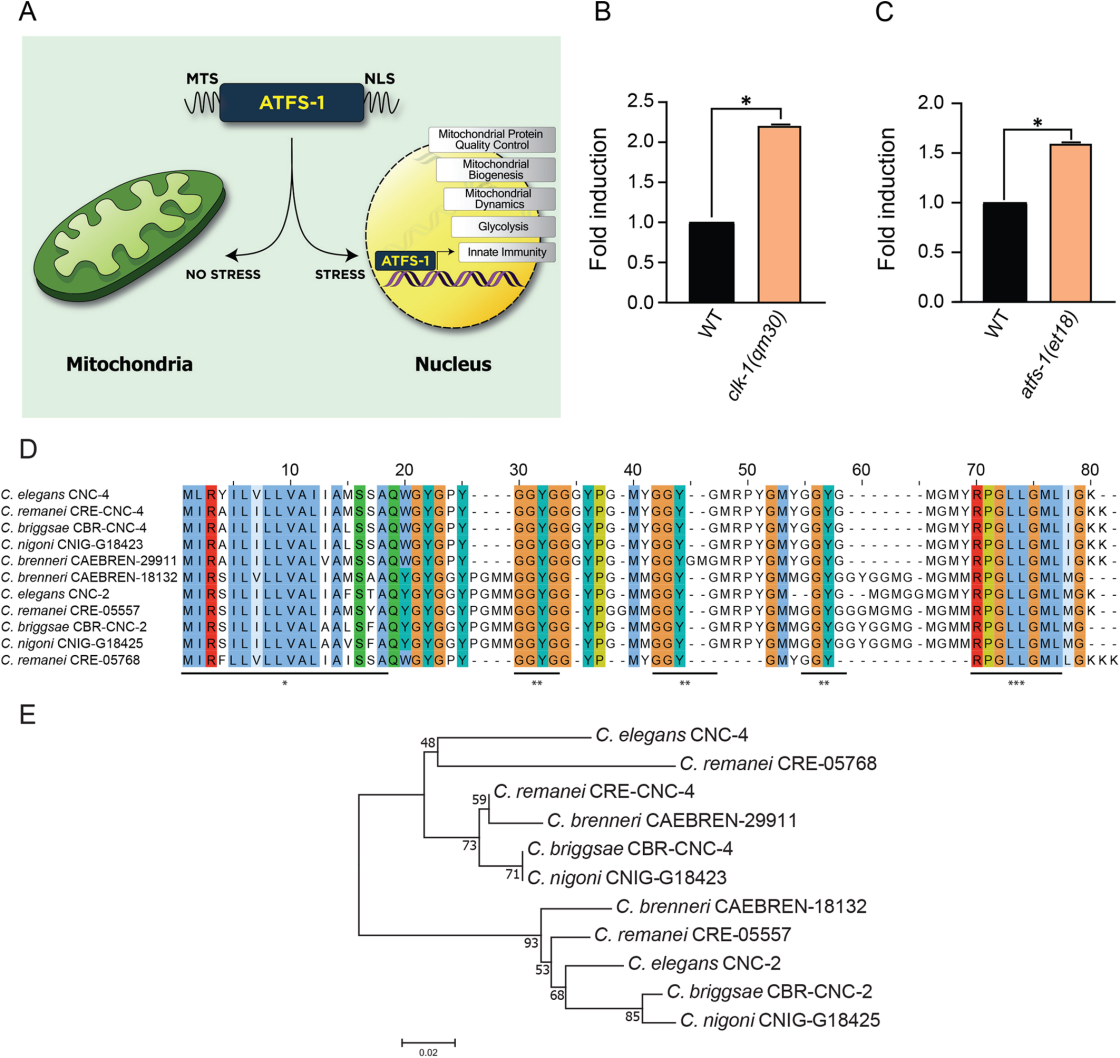
**The expression of *cnc-4* is upregulated during mitochondrial stress signaling.** (A) Schematic of the UPR^mt^ pathway in *C. elegans*. ATFS-1 contains both a mitochondrial targeting sequence (MTS) and nuclear localization sequence (NLS). Under healthy conditions, ATFS-1 enters mitochondria and is turned over proteolytically. During stress, import of ATFS-1 into mitochondria is reduced allowing the transcription factor to localize to the nucleus to regulate genes involved in mitochondrial recovery and innate immunity. (B) Quantitative PCR of *cnc-4* transcript levels in wild-type and *clk-1(qm30)* animals, *n*=3, ±s.d., *P<0.001 (Student's *t*-test). (C) Quantitative PCR of *cnc-4* transcript levels in wild-type and *atfs-1(et18)* animals; *n*=3, ±s.d., *P<0.001 (Student's *t*-test). (D) ClustalW multiple protein sequence alignment of CNC-4 with other conserved caenacin homologs. Colored regions denote conserved amino acid residues. Underlined regions: * denotes peptide signal sequence, ** denotes GGYG repeats, *** denotes conserved region of unknown function. (E) Phylogenetic analysis of CNC-4. The optimal tree with the sum of branch length=0.428 is shown. The percentage of replicate tree in which the associated taxa clustered together in the bootstrap test are shown next to the branches. The scale bar represents 0.02 substitutions per amino acid position.

Considering their significant importance, dysfunction to mitochondria needs to be efficiently mitigated so as to avoid cellular and organismal decline. The mitochondrial unfolded protein response (UPR^mt^) is one pathway that is activated during stress to mediate mitochondrial recovery (Qureshi et al., 2017). The mechanisms of UPR^mt^ regulation have largely been elucidated using the model organism *Caenorhabditis elegans* (Fig. 1A). The UPR^mt^ in *C. elegans* is regulated by the bZIP transcription factor ATFS-1 which possesses transport sequences to both the nucleus and mitochondria (Nargund et al., 2012). ATFS-1 is imported into healthy mitochondria and turned over by a protease-mediated mechanism (Nargund et al., 2012). However, mitochondrial dysfunction reduces protein import efficiency and consequently the entry of ATFS-1 into the organelle, resulting in the import of ATFS-1 into the nucleus (Nargund et al., 2012; Rolland et al., 2019). ATFS-1 regulates the transcription of genes with multiple roles in recovering damaged mitochondria, including mitochondrial chaperones and proteases that mediate protein homeostasis, as well as regulators of free radical detoxification and mitochondrial dynamics (Nargund et al., 2015, 2012). In addition to regulating genes that mitigate mitochondrial dysfunction, the UPR^mt^ also regulates innate immunity genes associated with defending the host against pathogenic agents. Notably, the UPR^mt^ is required for host survival during infection with bacterial pathogens such as *Pseudomonas aeruginosa* (Pellegrino et al., 2014). In addition, priming the host for the UPR^mt^ increases its resistance to pathogen infection that enhances survival (Jeong et al., 2017; Pellegrino et al., 2014).

With the knowledge that the UPR^mt^ protects the host during infection, we sought to identify genes that are regulated during the UPR^mt^ that possess direct antibacterial activity. Our search identified CNC-4, an antimicrobial peptide of the caenacin family. We found that *cnc-4* is induced transcriptionally during the UPR^mt^ and exhibits antimicrobial activity against a broad range of bacterial pathogens. Here, we have characterized the antimicrobial activity of CNC-4.

## RESULTS

### The antimicrobial peptide *cnc-4* is regulated by mitochondrial stress signaling

Activation of the UPR^mt^ protects the host during bacterial pathogen infection in an ATFS-1 dependent manner (Jeong et al., 2017; Pellegrino et al., 2014) (Fig. 1A). We sought to identify genes that might contribute to the antimicrobial activity of the UPR^mt^ by referencing previously conducted gene expression analysis datasets (Lin et al., 2016; Nargund et al., 2012). We found that the antimicrobial peptide *cnc-4* was transcriptionally induced during the UPR^mt^ in both gene expression datasets analyzed, suggesting the peptide may be a relevant innate immune mechanism that contributes to the antimicrobial activity of this stress response. Antimicrobial peptides are secreted factors that form part of the host's machinery to defend itself during infection. Their antibacterial nature relates to an ability to form disruptive pores in bacterial membranes, although alternative mechanisms have also been identified (Le et al., 2017). In addition, *cnc-4* was also found to be transcriptionally induced during various genetic perturbations that result in mitochondrial stress, including reduced function of OXPHOS components such as *cco-1*/COX5B (Tian et al., 2016), *clk-1*/COQ7 (Fischer et al., 2014) and *nuo-6*/NDUFB4 (Yee et al., 2014), as well as with knockdown of the mitochondrial chaperone *hsp-6*/mtHsp70 (Kim et al., 2016). We first confirmed that *cnc-4* transcript levels are increased during mitochondrial stress using the *clk-1(qm30)* loss-of-function mutant (Fig. 1B). Next, we validated that *cnc-4* was induced during the UPR^mt^ using an *atfs-1* gain-of-function mutant which contains a mutation in the mitochondrial targeting sequence of ATFS-1 that prevents its import into mitochondria, resulting in constitutive nuclear accumulation and UPR^mt^ activation (Rauthan et al., 2013). Consistently, *cnc-4* was upregulated in ATFS-1 gain-of-function animals (Fig. 1C), indicating that the gene encoding CNC-4 is transcriptionally induced during the UPR^mt^.

CNC-4 belongs to the caenacin family of antimicrobial peptides, which are phylogenetically related to the neuropeptide-like protein (NLP) family (Ewbank and Zugasti, 2011). Caenacins are largely restricted to nematodes and are known to be transcriptionally upregulated during fungal infection and exhibit anti-fungal properties (Couillault et al., 2004; Dierking et al., 2016).

A multiple sequence alignment of caenacins among different nematode species shows CNC-4 to be highly conserved amongst other members of the caenacin family and rich in glycine and aromatic amino acids (Fig. 1D; Table S1). A conserved and cleavable signal sequence is predicted from amino acids 1–18 of CNC-4, producing a mature peptide of 48 amino acids. After the signal sequence, a conserved motif of GGYG exists in all these peptides, followed by another highly conserved sequence RPGLLGML at the C-terminus. Interestingly, the GGYG motif is repeated throughout CNC-4 as well as in other caenacins, although the exact function of this motif is unknown. Phylogenetic analysis demonstrates that *C. elegans* CNC-4 forms a separate clade with CNC-4 proteins from *C. remanei* and *C. briggasae*, and three other caenacins from *C. remanei*, *C. brenneri* and *C. nigoni* (Fig. 1E). The other clade consists of CNC-2-related peptides from different nematodes including *C. elegans*.

### CNC-4 possesses broad-spectrum antibacterial activity

**Fig. 2.**
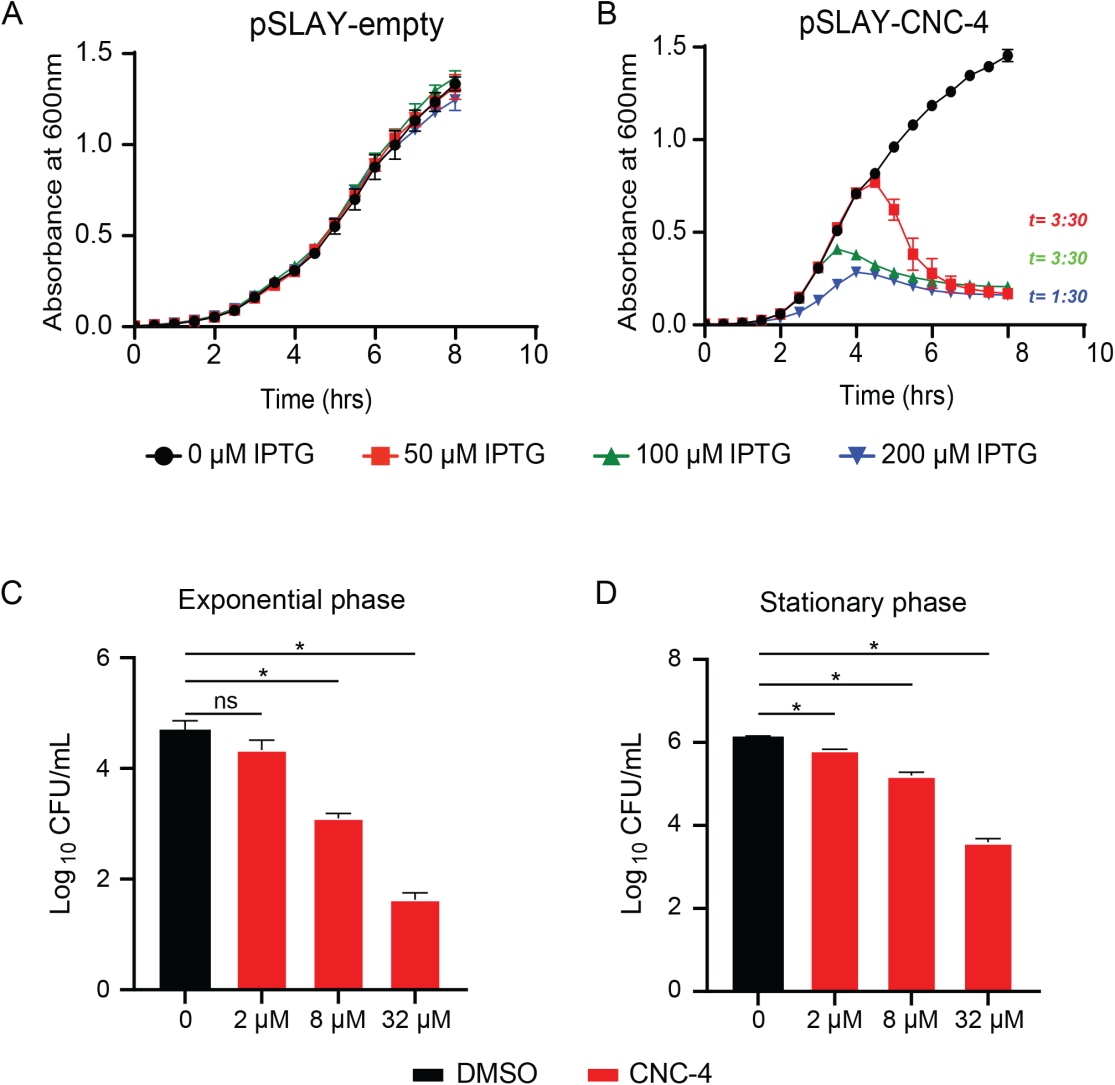
**CNC-4 demonstrates antibacterial activity.** (A,B) Growth of *E. coli* harboring SLAY (A) without CNC-4, or (B) with CNC-4. Cells were grown in increasing concentrations of IPTG and OD_600_ was measured every 0.5 h. *t*, time point after which reduction with statistical significance was achieved relative to IPTG control; *n*=3, ±s.d., *P*<0.05 (Student's *t*-test). (C,D) *E. coli* was treated with increasing doses of purified CNC-4 on cells isolated in (C) logarithmic growth phase or (D) stationary phase. DMSO was used as a control for relative comparison; *n*=3, ±s.d., *ns,* non-significant; *P<0.001 (Student's *t*-test).

Caenacins are known to respond to, and defend against, fungal infection in *C. elegans*. Whether they exhibit any antibacterial activity remained unknown. Therefore, we wished to assess potential antibacterial activity of CNC-4 by first using the surface localized antimicrobial display (SLAY) approach (Kazi et al., 2020; Tucker et al., 2018). Briefly, the SLAY IPTG-inducible expression plasmid includes the Lpp signal sequence, transmembrane domains of OmpA for outer membrane localization, and a flexible tether, followed by the peptide of interest as a C-terminal fusion, thus localizing it to the bacterial surface. The flexible tether enables the target peptide to interact with structures on the cell surface, within the outer membrane, periplasm and the inner membrane. Peptides with antimicrobial activity result in rapid cell death or growth inhibition. Antimicrobial activities of CNC-4 were assessed by SLAY using the *E. coli* K-12 strain W3110. As a negative control for our growth experiments, addition of IPTG inducer did not cause any growth defect in *E. coli* W3110 using the empty SLAY plasmid (Fig. 2A). However, CNC-4 expressed within the SLAY system caused substantial growth defects in *E. coli* W3110 in a dose-dependent manner (Fig. 2B).

To validate our findings, we chemically synthesized the active form of CNC-4 to measure its antibacterial activity in the absence of SLAY, as previously performed (Kazi et al., 2020; Tucker et al., 2018). We treated a subset of both Gram-negative and Gram-positive bacterial species with the purified CNC-4 peptide and calculated the minimum bactericidal concentration (MBC) as a reflection of its antibacterial activity. Purified CNC-4 exhibited potent antibacterial activity against Gram-negative bacteria including *E. coli, P. aeruginosa* and *Acinetobacter baumanii* with MBCs ranging from <2 µM to 8 µM (Table 1). In contrast, *Salmonella enterica* was relatively less susceptible to CNC-4 with a MBC of 128 µM (Table 1). Gram-positive bacteria were also impacted by CNC-4 treatment, including *Staphylococcus saprophyticus*, *Enterococcus faecalis* and *Staphylococcus epidermidis*, with MBC values ranging from 4 to 16 µM (Table 1), indicating that CNC-4 possesses broad-spectrum antibacterial activity.

**Table 1.**
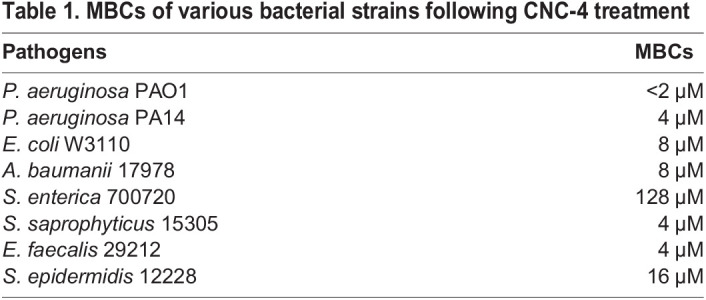
MBCs of various bacterial strains following CNC-4 treatment

We next evaluated whether bacterial growth phase impacted the antibacterial activity of CNC-4 using *E. coli* W3110 cells. We found that CNC-4 reduced the growth of log-phase *E. coli* W3110 cells in a concentration-dependent manner (Fig. 2C). To examine the susceptibility of stationary phase cells, overnight cultures of *E. coli* W3110 were treated with varying concentrations of CNC-4. Stationary phase *E. coli* W3110 were also susceptible to CNC-4-dependent killing, exhibited by reduced cell viability in a concentration-dependent manner (Fig. 2D). Therefore, bacterial growth phase stage does not impact CNC-4 antimicrobial action.

Next, we wondered whether physiological factors, including salt concentration and pH, affected the antibacterial activity of CNC-4. Salts can reduce the efficacy of antimicrobial peptides by reducing the electrostatic interaction of the peptide to the bacterial cell wall. Specifically, divalent cations such as Mg^2+^ bridge negatively charged functional groups of lipopolysaccharide located within the bacterial outer membrane, resulting in packing of lipids, while Na^+^ reduces antimicrobial peptide activity (Hancock, 1984; Kandasamy and Larson, 2006). We therefore measured the effects of monovalent (Na^+^) and divalent (Mg^2+^) cations on the activity of CNC-4 against *E. coli* W3110. We found that NaCl treatment led to decreases in the antibacterial activity of CNC-4 (Table 2). And, while low concentrations of MgCl_2_ had little effect on the antibacterial activity of CNC-4, higher concentrations (1 mM and 10 mM) led to increases in the MBC against *E. coli* W3110 (Table 2). We also examined the effects of pH on CNC-4 activity against *E. coli* W3110 and found that neutral and alkaline conditions were permissive to CNC-4 activity, whereas lower pH conditions were antagonistic (Table 2). Therefore, while monovalent and divalent salt concentrations may need to be considered with regards to the potency of CNC-4, pH may be of lesser concern since physiological conditions tend to be slightly more alkaline.

**Table 2.**
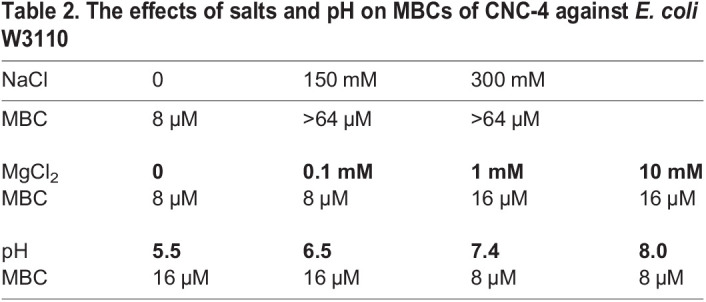
The effects of salts and pH on MBCs of CNC-4 against *E. coli* W3110

**Fig. 3.**
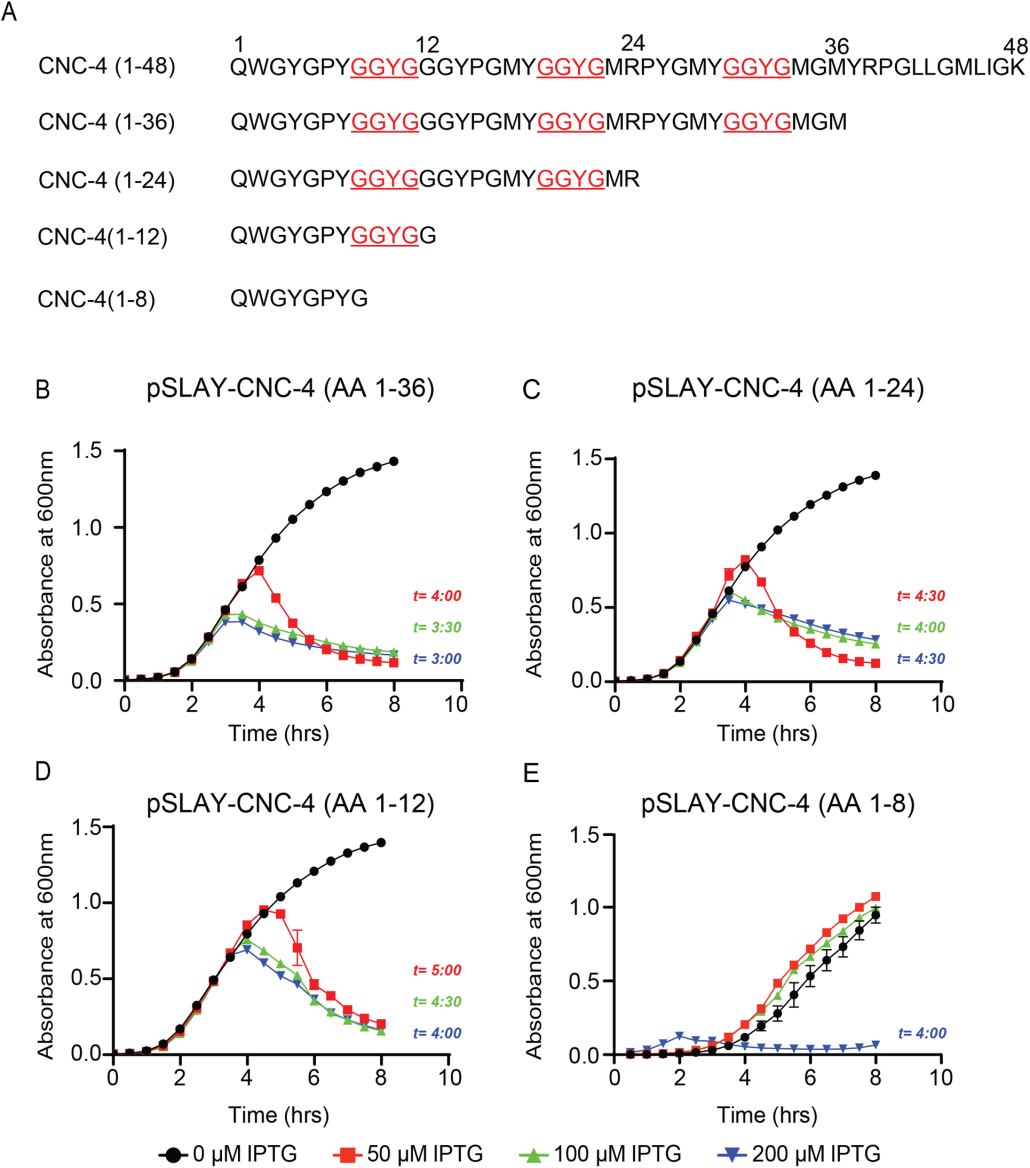
**The GGYG motif is necessary for CNC-4 antibacterial activity.** (A) Schematic of CNC-4 and the associated truncations used to determine its minimal activity domain. (B–E) Optical density plots measured from *E. coli* carrying SLAY-CNC-4 constructs, including (B) CNC-4(1–36), (C) CNC-4(1–24), (D) CNC-4(1–12) and (E) CNC-4(1–8) grown in increasing concentrations of IPTG. *t*, time point after which reduction with statistical significance was achieved relative to IPTG control; *n*=3, ±s.d., *P*<0.05 (Student's *t*-test).

Next, we wished to determine the minimal region of CNC-4 required for its antibacterial activity. We first created 12 amino acid truncations of CNC-4 and tested their antimicrobial activity using the SLAY system in *E. coli* W3110 cells (Fig. 3A). While several truncations were measured, deletion of amino acids 13–36 of the mature CNC-4 peptide had no measurable effect on growth (Fig. 3B–D). Interestingly, amino acids 1–12 of the mature CNC-4 contains the first GGYG motif, which is repeated three times and conserved amongst all caenacins (Fig. 1D). We therefore created an additional truncation specifically removing the GGYG repeat to assess its possible antimicrobial role. We observed that greater amounts of CNC-4(1–8) was required to disrupt bacterial growth compared to the mature CNC-4 or CNC-4(1–12) (Fig. 3E), suggesting that loss of the GGYG repeat reduces, but does not eliminate, CNC-4 antibacterial activity. Thus, the GGYG likely plays an important functional role in the antibacterial activity of CNC-4.

### CNC-4 increases bacterial membrane permeability

**Fig. 4.**
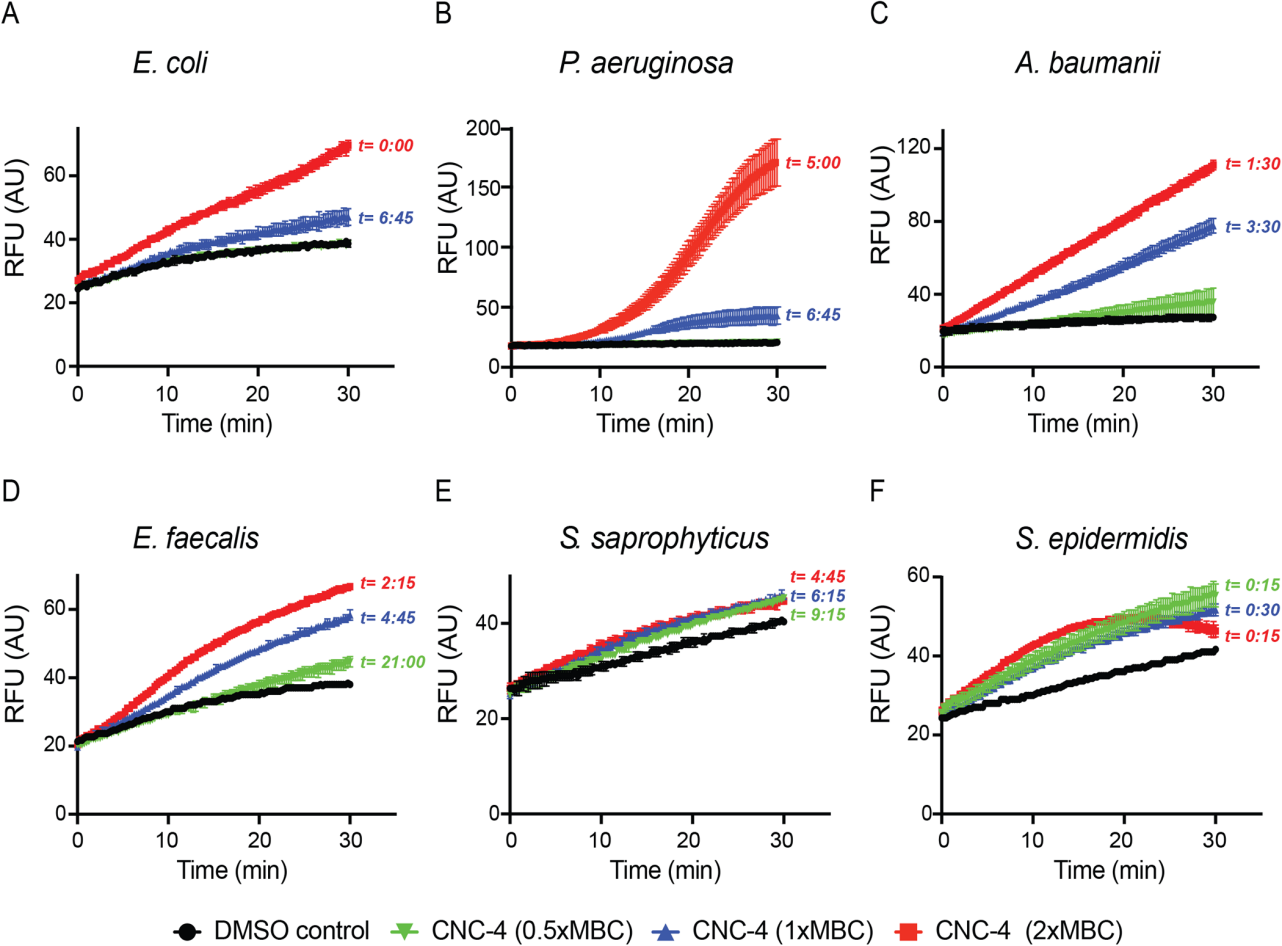
**CNC-4 exposure results in increased bacterial membrane permeability.** Effects of CNC-4 on outer membrane permeability in (A) *E. coli* W3110, (B) *P. aeruginosa*, (C) *A. baumannii*, (D) *E. faecalis*, (E) *S. saprophyticus* and (F) *S. epidermidis*, using the ethidium bromide influx assay (see Materials and Methods). *t*, time point after which statistical significance was achieved relative to DMSO control; *n*=3, ±s.d., *P*<0.05 (Student's *t*-test).

Next, we investigated the mechanism behind the antibacterial nature of CNC-4. We first examined the effect of CNC-4 on bacterial membrane permeability by quantifying the accumulation of intracellular ethidium bromide, as previously described (Kamischke et al., 2019; Kazi et al., 2020). Ethidium bromide is a fluorescent dye that intercalates DNA, but it is unable to enter Gram-negative bacteria due to their outer membrane that acts as a barrier. However, outer membrane perturbations, or loss of surface potential permits ethidium bromide entry, which can cross the cytoplasmic membrane and bind to its DNA target. We therefore quantified ethidium bromide accumulation in the presence or absence of CNC-4. Indeed, we found that CNC-4 increased the incorporation of ethidium bromide into Gram-negative bacteria including *E. coli* W3110, *P. aeruginosa* and *A. baumannii* in a dose-dependent manner (Fig. 4A–C). Gram-positive bacteria also displayed increases in membrane permeability in the presence of CNC-4, albeit to a lesser degree (Fig. 4D–F). Therefore, CNC-4 increases membrane permeability as part of its antibacterial mechanism.

**Fig. 5.**
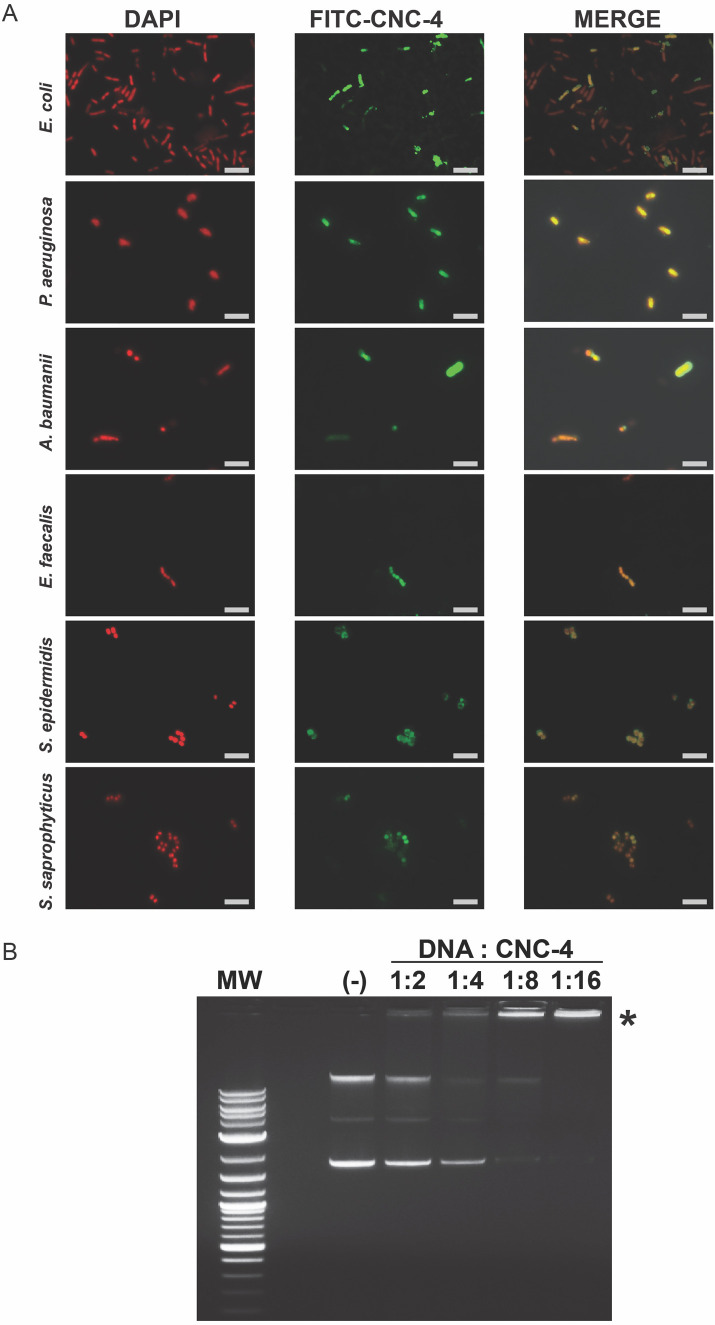
**CNC-4 localizes intracellularly and directly binds DNA.** (A) Localization of CNC-4 using FITC-labelled CNC-4 in *E. coli* W3110, *P. aeruginosa*, and *A. baumannii, E. faecalis*, *S. saprophyticus* and *S. epidermidis*. FITC-CNC-4, DAPI, and merged images were obtained by fluorescence microscopy. Scale bar is 5 µm. (B) Image of a representative gel electrophoresis of plasmid DNA/CNC-4 combinations at the indicated mass ratios. MW, molecular weight ladder (1 kb DNA ladder; Promega); (-), plasmid DNA/DMSO negative control; * denotes region in which DNA failed to substantially migrate for DNA:CNC-4 ratios 1:4, 1:8 and 1:16.

We also examined the localization of FITC-CNC-4 using fluorescence microscopy. We found that FITC-CNC-4 accumulated within the cytoplasm of both Gram-negative and Gram-positive bacteria based on co-localization with DAPI-stained DNA (Fig. 5A). Consistently, intracellular localization of antimicrobial peptides, which result in increased membrane permeabilization, has been observed before (Farkas et al., 2017). While this suggests that the antimicrobial mode of action of CNC-4 may occur at least in part intracellularly, it is probable that CNC-4 resides at the membrane transiently, leading to increased bacterial membrane permeability and accumulation within the cytoplasm.

Since we observed intracellular accumulation of CNC-4, we also investigated other possible molecular targets of this peptide. Antimicrobial peptides can also promote their antibacterial activity by directly binding bacterial DNA (Le et al., 2017). To examine whether CNC-4 exhibited DNA binding properties, we incubated plasmid DNA with increasing amounts of CNC-4 peptide and then assessed migratory shifts following electrophoresis. Interestingly, increasing amounts of CNC-4 prevented plasmid DNA migration during electrophoresis (Fig. 5B), suggesting that CNC-4 possesses DNA binding activity.

### CNC-4 does not show cytotoxicity against mammalian epithelial cells

**Fig. 6.**
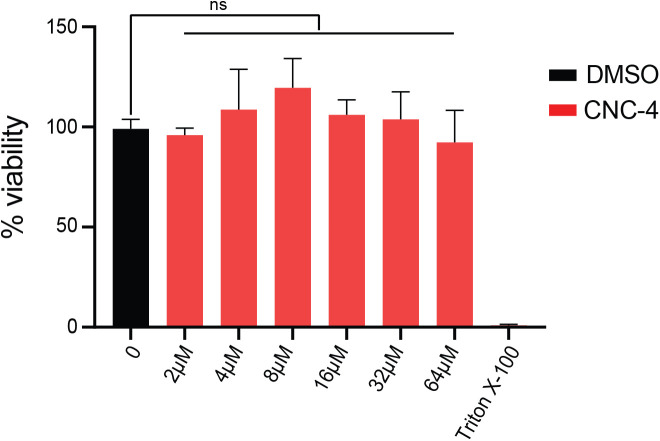
**CNC-4 does not exhibit toxicity to mammalian epithelial cells.** Quantification of CNC-4 toxicity to MODE-K cells using the MTT assay (see Materials and Methods). MODE-K cells at ∼50,000 cells/well of density were treated with the indicated concentrations of purified CNC-4 peptide. Triton X-100 was used a positive control which leads to complete loss of cell viability; *n*=3, ±s.d.; *ns,* non-significant (Student's *t*-test).

Finally, we examined whether CNC-4 exhibited any toxicity towards mammalian cells using the mouse derived intestinal cell line MODE-K as previously performed (Kazi et al., 2020). To measure cytotoxicity, we employed the MTT [3-(4,5-dimethylthiazol-2-yl)-2,5-diphenyltetrazolium bromide] assay which measures the ability of viable cells to convert MTT to an insoluble formazan product. Our results indicated that MODE-K cells tolerate CNC-4 at concentrations up to 64 µM (Fig. 6), which is well above the MBC for several pathogens tested (Table 1). Therefore, CNC-4 may have the potential to be used as an antimicrobial agent with the added benefit of reduced toxicity to the host.

## DISCUSSION

In conclusion, while mitochondrial stress signaling pathways such as the UPR^mt^ have documented benefits in increasing host resistance during pathogen infection, the effectors that mediate this protection have largely been unresolved. We have identified the antimicrobial peptide CNC-4 as one such effector, demonstrating notable activity against several clinically important bacterial pathogens. We showed that CNC-4 exhibits antibacterial activity presumably through a mechanism involving increased membrane permeability and possibly DNA binding mechanisms. In addition, CNC-4 exhibits low cytotoxic activity against a mammalian cell line. Together, our data suggest that the nematode-originating CNC-4 may have potential applications therapeutically against challenging bacterial infections.

It was previously shown that the UPR^mt^ regulates innate immunity gene expression to protect the host against infection (Amin et al., 2020; Jeong et al., 2017; Mahmud et al., 2020; Pellegrino et al., 2014; Shao et al., 2020). The *cnc-4* gene is transcriptionally induced during mitochondrial stress as part of the UPR^mt^ protective pathway, however the exact mechanism for this UPR^mt^-mediated *cnc-4* induction is currently unclear. One possibility is that the main UPR^mt^ transcription factor ATFS-1 directly regulates the expression of *cnc-4*. However, we did not detect any obvious ATFS-1 binding sites (Nargund et al., 2015) in the *cnc-4* promoter. Also, *cnc-4* was not identified as a direct target of ATFS-1 using chromatin immunoprecipitation (Nargund et al., 2015). Therefore, it is likely that ATFS-1 regulates *cnc-4* expression indirectly via an as-of-yet unknown regulator. It was previously found that the mitochondrial chaperone HSP-60, which is transcriptionally induced during the UPR^mt^, can stimulate innate immunity through physical association with SEK-1/MAP kinase kinase 3, a critical component of the p38 MAP kinase innate immunity pathway in *C. elegans* (Jeong et al., 2017). Thus, one possibility is that p38 MAP kinase signaling mediates the upregulation of *cnc-4* during the UPR^mt^. However, gene expression for the related caenacin *cnc-2* was found to be induced during infection with the fungus *Drechmeria coniospora* in a p38 MAP kinase-independent manner (Zugasti and Ewbank, 2009). Instead, *cnc-2* gene induction was found to rely on non-canonical TGF-β signaling (Zugasti and Ewbank, 2009). Therefore, it is possible that the UPR^mt^ and non-canonical TGF-β signaling converge to regulate *cnc-4* gene expression during stress.

A discernible feature of CNC-4 is the high representation of glycine residues in the mature peptide (40% of all amino acids), a characteristic feature of members of the caenacin family (Couillault et al., 2004). While the exact function of the glycine residues is not known, it is predicted to increase flexibility to the peptide's structure (Pelegrini et al., 2008). Another discernible feature of CNC-4 is the presence of a GGYG repeat that we showed to be necessary for its antimicrobial activity. We currently do not know the functional significance of this repeat sequence but interestingly, it is also found in a crustacean antimicrobial peptide that exhibits specific activity against Gram-positive bacteria (Imjongjirak et al., 2011). Other glycine-rich antimicrobial peptides have also been discovered that exhibit activities against fungi and/or bacteria (both Gram-negative and Gram-positive) (Bulet et al., 1991; de Jesus Oliveira et al., 2019; Lorenzini et al., 2003; Lu and Chen, 2010; Sousa et al., 2009; Sperstad et al., 2009; Verdon et al., 2016; Xie et al., 2020). While the caenacins were previously shown to possess anti-fungal properties, our study clearly demonstrates an antibacterial activity for CNC-4 against both Gram-negative and Gram-positive bacteria. Interestingly, another member of the *C. elegans* caenacin family, CNC-2, mediates anti-fungal defense specifically (Zehrbach et al., 2017), suggesting these peptides exhibit variable antimicrobial activities.

Our study has also elucidated the mechanism of action for the caenacin CNC-4. Here, we have shown that CNC-4 increases membrane permeability in both Gram-negative and Gram-positive bacteria. Supporting this finding, we also showed that fluorescently-labelled CNC-4 accumulated intracellularly in both Gram-negative and Gram-positive bacteria. Lastly, we discovered that CNC-4 has the capability of binding DNA, suggesting that its buildup within the cell may have functional significance. Interestingly, a glycine-rich antimicrobial peptide (YD1) was isolated from *Bacillus amyloliquefaciens* that displayed cell-penetrating capabilities and also DNA-binding properties (Rahman et al., 2017). In contrast to CNC-4, however, YD1 was able to penetrate bacterial membranes without the formation of disruptive pores (Rahman et al., 2017). Similarly, CF-14, an antimicrobial peptide isolated from catfish epidermal mucus, was able to access the inside of bacterial cells and bind DNA (Li et al., 2019). Lastly, the cell-penetrating synthetic antimicrobial peptide analog P7 was shown to bind bacterial DNA and inhibit cell division (Li et al., 2015). Whether CNC-4 displays cell penetrating abilities in addition to its capacity to form disruptive pores is not known. The functional significance of CNC-4's ability to bind DNA is also unclear. However, other antimicrobial peptides have been found to bind DNA resulting in cessation of DNA replication. One such example is indolicidin, an antimicrobial peptide that prevents DNA unwinding, resulting in repression of DNA replication and transcription (Ghosh et al., 2014; Subbalakshmi and Sitaram, 1998). Further mechanistic examination into the antimicrobial nature of CNC-4 is thus warranted.

## MATERIALS AND METHODS

### *C. elegans* strains and culturing

*C. elegans* were obtained from the *Caenorhabditis* Genetics Center and cultured as described previously using standard Nematode Growth Medium (Brenner, 1974). Worms were cultured at 20°C and fed *E. coli* OP50.

### Bacterial strains, antibiotics and CNC-4 peptide

Strains and plasmids used in this study are listed in Tables S2 and S3. The concentration of antibiotics used were: ampicillin (50 µg/ml) and tetracycline (5–10 µg/ml). Bacterial cultures were grown at 37°C with shaking at 280 rpm. CNC-4 peptide was synthesized commercially (GenScript) and dissolved in 100% DMSO to a concentration of 10 mg/ml and diluted to the appropriate concentration thereafter, as performed previously (Kazi et al., 2020; Tucker et al., 2018).

### Quantitative PCR (qPCR) of cnc-4 expression

*C. elegans* total RNA was harvested by using Direct-zol™ RNA MiniPrep Plus kit as per manufacturer's recommendations. cDNA was synthesized using the Bio-Rad Superscript kit according to the manufacturer's instructions. qPCR of *cnc-4* was performed using primers ACAATGGGGCTACGGTCCATAT and ACTTTCCAATGAGCATTCCGAGGA, with amplification of the housekeeping gene *act-3* as a reference using primers ATCCGTAAGGACTTGTACGCCAAC and CGATGATCTTGATCTTCATGGTTC.

### CNC-4 protein sequence analysis

The CNC-4 protein sequence was blasted against UniProt's UniProtKB reference proteomes plus Swiss-Prot database using BLASTP and the top ten non-redundant hits were identified. Protein sequences were gathered, and multiple sequence alignment of those proteins sequences was performed using the ClustalW tool in BioEdit. The alignment file was imported into JalView to construct the final alignment figure. For generation of the phylogenetic tree, the same alignment file was imported into MEGA 7.0 software and the tree generated using the Neighbor-Joining method using 1000 bootstraps. All positions containing gaps and missing data were eliminated.

### SLAY expression plasmid construction

The CNC-4 cDNA was amplified using primers CGCGGTACCAGTCAAGAGCCTGC and AAAGTCGACTTACTTTCCAATGAGCATTCCGAGGAGCCCTGGGCGGTACATTCCCATTCCGTAGCCACCGTACATTCCATATGGACGCATACCGTATCCGCCATACATTCCAGGGTATCCACCACCATACCCGCCATATGGACCGTAGCCCCATTGTCCTCCGATACCCGCAGCTG, and pMMB-cecropin (Tucker et al., 2018) was used as a template. The amplicon was digested with KpnI and SalI and cloned into the pMMB-tet plasmid yielding pMMB-tet-cnc-4. The construct was transformed into the *E. coli* W3110 strain by chemical transformation. CNC-4 truncated cDNA fragments were amplified using the forward primer ACAGAATTCAGGAGGAAACGATGAAA and the following reverse primers: TTTGTCGACTTACATTCCCATTCCGTAGCC (CNC-4_1–36), TTTGTCGACTTAACGCATACCGTATCCGCC (CNC-4_1–24), TTTGTCGACTTAACCACCATACCCGCCATA (CNC-4_1–12) and TTTGTCGACTTAGCCATATGGACCGTAGCCCCATTG (CNC-4_1–8), using pMMB-tet-cnc-4 as a template) and cloned into EcoRI and SalI sites of pMMB67EH. Each construct was verified by DNA sequencing before transforming into *E. coli* W3110.

### Bacterial growth determination using SLAY display expression system

Growth curves using SLAY were done as done previously described (Kazi et al., 2020; Tucker et al., 2018). Overnight cultures of *E. coli* W3110 were diluted to OD_600_ of ∼0.05 and grown to an OD_600_ of 0.6–0.8 representative of exponentially growing cells or overnight for stationary phase cells. Bacterial cultures were diluted to an OD_600_ of 0.01 and loaded into 96-well plates with increasing concentration of IPTG. Bacterial growth was monitored using a BioTek SYNERGY neo2 multi-mode plate reader every 30 min for 8 h. Growth curves were carried out at least three times in triplicate and plotted using GraphPad.

### Determination of MBC

MBCs were performed as previously described (Kazi et al., 2020; Tucker et al., 2018), with slight modifications. The MBC was defined as the minimum concentration of the CNC-4 peptide, which resulted in a three-log growth reduction of the initial inoculum. The MBC assays were carried out in bovine serum albumin (BSA)-acetic acid media (Tris-medium assay). Briefly, cells were grown to an OD_600nm_ of 0.6–0.8, harvested with centrifugation at 3000 rpm for 5 min and washed at least twice with Tris-media comprised of 10 mM Tris-base (pH 7.4), 50 mM NaCl, 0.2% glucose. The OD_600nm_ of the cultures were adjusted to 0.004 in Tris-media. CNC-4 peptide was prepared in BSA-acetic acid media (0.2% BSA, 0.01% acetic acid) and then added to the cultures at varying concentrations. Solutions were mixed thoroughly and incubated in polypropylene 96-well plates for 18–24 h at 37°C. DMSO was used as a control for each concentration of CNC-4 peptide. 5 µl of each overnight culture was spotted on LB agar media and incubated overnight. The concentration of CNC-4 peptide that resulted in no visible growth was recorded as the MBC.

### Membrane permeability assay

Permeability assays were performed as previously described (Kamischke et al., 2019; Kazi et al., 2020). Bacterial cells were grown to mid-log phase followed by washing with Tris-media. Bacteria were then diluted to OD_600_ of 0.3/ml. CNC-4 peptide was added to a concentration equivalent to 0.5×MBC, 1×MBC and 2×MBC values for each strain. Ethidium bromide was added to a final concentration of 6 µM followed by measurement of fluorescence using a BioTek SYNERGY neo2 multi-mode plate reader at 545 nm excitation and 600 nm emission wavelengths.

### Fluorescence microscopy

The CNC-4 full-length peptide was labelled with FITC (fluorescein isothiocyanate) at its N-terminus by GenScript. Bacterial strains were grown to mid-log phase, harvested and washed at least twice with Tris-media before treatments with 1 µM FITC-CNC-4. About 10^7^ CFUs of cells were used for peptide treatment. Cells were treated for the specified amount of time and washed with Tris-media. Bacterial cells were then stained with DAPI (1 µg/ml) and images were acquired using a Zeiss Axioimager.Z2 fluorescence microscope.

### CNC-4 DNA-binding assay

The interaction of CNC-4 with plasmid DNA was assessed using the gel retardation assay (Chen et al., 2013). 500 ng of pBlueScript SK(-) plasmid was combined with varying amounts of purified CNC-4 peptide. DMSO was used as a control for the peptide solvent. Each mixture was incubated at 37°C for 1 h and separated using standard gel electrophoresis.

### MTT assay for the assessment of cell viability

MTT assay was carried out using the MTT Cell Growth Assay kit as described by the manufacturer (EMD Millipore), as previously described (Kazi et al., 2020). Mouse MODE-K epithelial cell line (gift from Dr. Jason Kubinak, University of South Carolina, USA) were used at sub-confluent (∼10,000 cells/well) and confluent (∼50,000 cells/well) cell densities in RPMI media with 10% FBS. MODE-K cells were treated with increasing concentrations of dimethyl sulfoxide (DMSO) or CNC-4 peptide for 24 h. Cells were then treated with fresh media containing 0.45 mg/ml 3-(4,5-dimethyl-2-thiazolyl0-2,5-diphenyltetrazolium bromide (MTT) (Sigma-Aldrich) and incubated at 37°C for 4 h. The liquid was then removed and 100 µl of DMSO added to each well. The plate was incubated at room temperature with shaking for 1 h and the absorbance was measured at 570 nm. The DMSO vehicle control represents the volume equivalent used for 64 µM CNC-4.

## Supplementary Material

Supplementary information
