# Supplementary material for: A nematode-derived, mitochondrial stress signaling-regulated peptide exhibits broad antibacterial activity
Source: Biol Open. 2021 May 20;10(5):bio058613. doi: 10.1242/bio.058613 (PMC8181894; doi:10.1242/bio.058613)
Supplement: Supplementary information [file biolopen-10-058613-s1.pdf]

## SUPPLEMENTARY TABLES

### Table S1. The physical properties of CNC-4.

| | |
|---|---|
| **Amino acid sequence** | QWGYGPYGGYGGGYPGMYGGYGMRPYGMYGG YGMGMYRPGLLGMLIGK |
| **Net charge (pH 7)/pI** | 3/9.52 |
| **Theoretical MW,mass** | 5116.9, 7.1kD |
| **Polar/non-polar residues (%)** | 0.067 |
| **Hydrophobic face residues** | MGYMGYYYYPYMGYYGPG |
| **Hydrophobicity<H>** | 0.537 |
| **Hydrophobic moment< μH>** | 0.099 |

Biology Open • Supplementary information

**Table S2. List of plasmids used in this study.**

| Plasmids | Comments | References |
|---|---|---|
| pMMB67EHTet | pMMB67EH with tetracycline resistance gene, (Tet) | PMID: 32591801 |
| pMMB67EH with lpp ompA cecropin P1 | pMMB67EH with lpp-OmpA-tether (Amp) | PMID: 29307492 |
| pMIK102 (SLAY) | pMMB67EHTet with lpp-OmpA-tether, (Tet) | PMID: 32591801 |
| SLAY-CNC-4 (1-48) | Full length CNC-4 cloned into pMIK102 | This study |
| SLAY-CNC-4(1-36) | 1-36 residues CNC-4 cloned into pMIK102 | This study |
| SLAY-CNC-4(1-24) | 1-24 residues CNC-4 cloned into pMIK102 | This study |
| SLAY-CNC-4(1-12) | 1-12 residues CNC-4 cloned into pMIK102 | This study |
| SLAY-CNC-4(1-8) | 1-8 residues CNC-4 cloned into pMIK102 | This study |

Biology Open • Supplementary information

**Table S3. List of bacterial strains used in this study.**

| Strains | Source |
|---|---|
| *P. aeruginosa* PAO1 | Joao Xavier (MSKCC) |
| *P. aeruginosa* PA14 | Joao Xavier (MSKCC) |
| *E. coli* W3110 | *E. coli* Genetic Stock Center (Yale) |
| *A. baumanii* | ATCC17978 |
| *S. enterica* subsp. *enterica* serovar Typhimurium | ATCC 700720 |
| *Staphylococcus saprophyticus* | ATCC 15305 |
| *Enterococcus faecalis* | ATCC 29212 |
| *Staphylococcus epidermidis* | ATCC 12228 |

Biology Open • Supplementary information
